# ^68^Ga-NODAGA-Exendin-4 PET/CT Improves the Detection of Focal Congenital Hyperinsulinism

**DOI:** 10.2967/jnumed.121.262327

**Published:** 2022-02

**Authors:** Marti Boss, Christof Rottenburger, Winfried Brenner, Oliver Blankenstein, Vikas Prasad, Sonal Prasad, Paolo de Coppi, Peter Kühnen, Mijke Buitinga, Pirjo Nuutila, Timo Otonkoski, Khalid Hussain, Maarten Brom, Annemarie Eek, Jamshed Bomanji, Pratik Shah, Martin Gotthardt

**Affiliations:** 1 Department of Medical Imaging, Radboud University Medical Centre, Nijmegen, The Netherlands;; 2 Division of Nuclear Medicine, University Hospital Basel, Basel, Switzerland;; 3 Institute of Nuclear Medicine, University College London, London, United Kingdom;; 4 Department of Nuclear Medicine, Charité–Universitätsmedizin, Berlin, Germany;; 5 Institute for Experimental Pediatric Endocrinology, Charité–Universitätsmedizin, Berlin, Germany;; 6 Department of Nuclear Medicine, University Hospital of Ulm, Ulm, Germany;; 7 Berlin Experimental Radionuclide Imaging Center, Charité–Universitätsmedizin, Berlin, Germany;; 8 Department of Pediatric Surgery, Great Ormond Street Hospital for Children, NHS Foundation Trust, London, United Kingdom;; 9 Department of Endocrinology, Turku University Hospital, Turku, Finland;; 10 Turku PET Center, University of Turku, Turku, Finland;; 11 Stem Cells and Metabolism Research Program, Faculty of Medicine, University of Helsinki, Helsinki, Finland;; 12 Children’s Hospital, University of Helsinki and Helsinki University Hospital, Helsinki, Finland;; 13 Department of Pediatric Medicine, Division of Endocrinology, Sidra Medical and Research Center, Doha, Qatar;; 14 Pediatric Endocrinology Department, Great Ormond Street Hospital for Children, NHS Foundation Trust, London, United Kingdom; and; 15 Department of Pediatric Endocrinology, Royal London Children’s Hospital, Bart’s Health NHS Trust, London, United Kingdom

**Keywords:** congenital hyperinsulinism, focal CHI, diagnostic imaging, ^68^Ga-NODAGA-exendin-4 PET/CT, ^18^F-DOPA PET/CT

## Abstract

Surgery with curative intent can be offered to congenital hyperinsulinism (CHI) patients, provided that the lesion is focal. Radiolabeled exendin-4 specifically binds the glucagonlike peptide 1 receptor on pancreatic β-cells. In this study, we compared the performance of ^18^F-DOPA PET/CT, the current standard imaging method for CHI, and PET/CT with the new tracer ^68^Ga-NODAGA-exendin-4 in the preoperative detection of focal CHI. **Methods:** Nineteen CHI patients underwent both ^18^F-DOPA PET/CT and ^68^Ga-NODAGA-exendin-4 PET/CT before surgery. The images were evaluated in 3 settings: a standard clinical reading, a masked expert reading, and a joint reading. The target (lesion)-to-nontarget (normal pancreas) ratio was determined using SUV_max_. Image quality was rated by pediatric surgeons in a questionnaire. **Results:** Fourteen of 19 patients having focal lesions underwent surgery. On the basis of clinical readings, the sensitivity of ^68^Ga-NODAGA-exendin-4 PET/CT (100%; 95% CI, 77%–100%) was higher than that of ^18^F-DOPA PET/CT (71%; 95% CI, 42%–92%). Interobserver agreement between readings was higher for ^68^Ga-NODAGA-exendin-4 than for ^18^F-DOPA PET/CT (Fleiss κ = 0.91 vs. 0.56). ^68^Ga-NODAGA-exendin-4 PET/CT provided significantly (*P* = 0.021) higher target-to-nontarget ratios (2.02 ± 0.65) than did ^18^F-DOPA PET/CT (1.40 ± 0.40). On a 5-point scale, pediatric surgeons rated ^68^Ga-NODAGA-exendin-4 PET/CT as superior to ^18^F-DOPA PET/CT. **Conclusion:** For the detection of focal CHI, ^68^Ga-NODAGA-exendin-4 PET/CT has higher clinical sensitivity and better interobserver correlation than ^18^F-DOPA PET/CT. Better contrast and image quality make ^68^Ga-NODAGA-exendin-4 PET/CT superior to ^18^F-DOPA PET/CT in surgeons’ intraoperative quest for lesion localization.

## Visual Abstract

**Figure:**
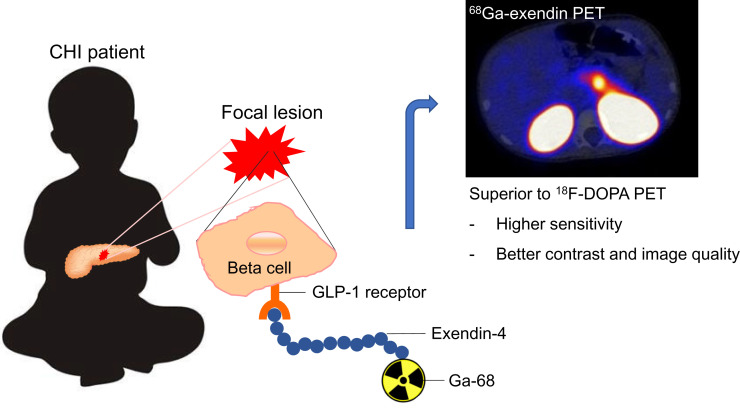

Congenital hyperinsulinism (CHI) is the most common cause of persistent and recurrent hypoglycemia in neonates. It occurs with an incidence of 1 in 35,000–40,000 births (*1*). CHI often presents in neonates as poor feeding, seizures, jitteriness, hypotonia, apnea, cyanosis, hypothermia, or a hypoglycemia-induced life-threatening event (*2*). CHI can also manifest in infancy or childhood and, in rare cases, even in adolescents or young adults (*3*). To avoid brain injury, early diagnosis and proper treatment of CHI are crucial. The diagnosis of CHI is based on clinical findings and hypoglycemic events, combined with inappropriately high insulin or C-peptide levels or low insulinlike growth factor– binding protein 1 levels (*4*,*5*). In diffuse CHI, which accounts for 60%–70% of all cases, there is diffuse involvement of the pancreatic β-cells, with enlarged hyperfunctioning cells that have abnormally large nuclei and abundant cytoplasm (*6*,*7*). This subform is caused by recessive or dominant mutations in the *ABCC8* or *KCNJ11* genes, encoding for the β-cell adenosine triphosphate– sensitive potassium channels. Diffuse CHI is treated primarily with medication, such as octreotide and diazoxide. However, many patients with recessive mutations in the *ABCC8* and *KCNJ11* genes are unresponsive to this therapy, and near-total pancreatectomy may then be the only option to avoid devastating hypoglycemia. Even after such an invasive procedure, some children present with recurring hypoglycemia, requiring further treatment with medication or even reoperation (*8*).

Focal CHI accounts for 30%–40% of all CHI cases associated with the adenosine triphosphate–sensitive potassium channel genes. This form is characterized by focal adenomatous islet cell hyperplasia caused by the concurrence of a paternal mutation in the *ABCC8* or *KCNJ11* gene and somatic loss of heterozygosity of the maternal chromosome 11p15 region within a limited pancreatic region (*9*,*10*). Because there is involvement of only a specific pancreatic area, focal CHI can be treated successfully by partial pancreatectomy or limited lesionectomy, which can cure the disease in cases of complete removal of the lesion (*7*). Since focal CHI can be treated with much less invasive surgery than diffuse CHI, correct differentiation between these subforms is of great importance. Also, precise presurgical localization of the focal lesion is important for correct surgical planning and optimization of surgical outcomes. If the lesion resides in the body or tail of the pancreas, a minimally invasive, laparoscopic, procedure may be performed (*11*).

The current standard imaging technique for noninvasive detection of focal CHI is ^18^F-DOPA PET/CT (*12*). Because this technique has a sensitivity of only 85%–89% for the detection of focal CHI (*13*), focal lesions are still missed in some cases. In this study, we used a new radiotracer based on the peptide exendin-4, which binds with high affinity specifically to the glucagonlike peptide 1 receptor expressed on pancreatic β-cells (*14*). ^68^Ga-labeled exendin has been shown to detect insulinomas with high sensitivity (*15*,*16*). The specific tracer used in the current study, ^68^Ga-NODAGA-exendin-4, is being assessed in a large prospective trial for insulinoma imaging (NCT03189953). We have analyzed the data of patients with CHI who underwent both ^18^F-DOPA PET/CT and ^68^Ga-NODAGA-exendin-4 PET/CT to compare the effectiveness of these 2 imaging techniques for the detection and localization of focal CHI.

## MATERIALS AND METHODS

### Study Design and Patients

This prospective multicenter study (NCT03768518) included consecutive eligible patients at the Great Ormond Street Hospital in the United Kingdom (7 patients) and at the Radboud University Medical Centre in The Netherlands (1 patient). Patients were recruited directly by these centers and by referral from several tertiary centers across Europe.

Patients were enrolled with biochemically proven, endogenous CHI who were unresponsive to medical treatment and qualified for ^18^F-DOPA PET/CT on the basis of mutation analysis (no genetically proven diffuse CHI based on a homozygous or compound heterozygous *ABCC8/KCNJ11* mutation). Exclusion criteria were renal insufficiency (creatinine clearance < 40 mL/min) and evidence of malignancies other than insulin-producing lesions. The study was approved by the local institutional review board of both participating institutes. The parents of all included patients provided written informed consent in accordance with provisions of the Declaration of Helsinki.

In addition, real-world evidence data from CHI patients diagnosed and treated at the Charité University Hospital in Germany (11 patients) were analyzed in accordance with national drug regulations. Parents of these patients provided written informed consent to the use of the new radiotracer.

### Procedures

In all patients, ^18^F-DOPA PET/CT was performed according to the local guidelines for focal-CHI detection. ^18^F-DOPA (3 MBq/kg; lower limit, 40 MBq) was injected as a slow bolus over 1 min. Patients had not fasted and were not pretreated with carbidopa. A PET/CT acquisition at 1 bed position for 10 min was started 20 min after tracer injection. Depending on the assessment of the first scan, additional PET acquisitions were performed at 40 or 60 min after tracer injection.

For ^68^Ga-NODAGA-exendin-4 PET/CT, a 1.6 ± 0.1 MBq/kg dose of the tracer (lower limit, 20 MBq), corresponding to a peptide dose of maximally 0.08–0.12 µg/kg (lower limit, 1.4 µg), was injected intravenously as a slow bolus over 5 min. Details on radiopharmaceutical preparation are provided in the supplemental materials. The patients had fasted for 1 h before tracer injection to reduce endogenous glucagonlike peptide 1 production. Blood glucose levels were monitored before tracer injection and at least at 5, 10, 15, 30, 60, 90, and 120 min after tracer injection. Since blood glucose levels may decrease after ^68^Ga-NODAGA-exendin-4 injection, they were closely monitored. Intravenous glucose injection with a case-specific infusion rate was given to all patients to manage glucose levels during the procedure.

PET acquisition methods varied because of differences in the institutional standard of care for CHI patients. Details on ^68^Ga-NODAGA-exendin-4 PET/CT and PET/MRI acquisition procedures for all centers and reconstruction parameters are given in Supplemental Table 1 (supplemental materials are available at http://jnm.snmjournals.org). At the Great Ormond Street Hospital, a protocol has been developed for ^18^F-DOPA PET/CT/CT to be performed under oral sedation with chloral hydrate in children with CHI to avoid general anesthesia (Sarah Kiff et al., unpublished data, 2021). This protocol was adopted for ^68^Ga-NODAGA-exendin-4 PET/CT acquisitions at the Great Ormond Street Hospital.

At the Radboud University Medical Centre, 1 patient was included. Scans there were performed without anesthesia with a vacuum mattress.

Real-world evidence from an institutional database of 11 CHI patients who underwent ^68^Ga-NODAGA-exendin-4 PET/CT for diagnostic purposes at the Charité University Hospital were included retrospectively. Younger children were imaged while receiving inhalation anesthesia with isoflurane under the supervision of an anesthesiologist; older children were able to undergo the procedure without sedation.

### Evaluation

Histologic evaluation and clinical outcome (normalization of blood glucose levels after surgery) were used as a reference standard. ^18^F-DOPA PET/CT and ^68^Ga-NODAGA-exendin-4 PET/CT scans were clinically read at the site of patient inclusion by nonmasked observers. The clinical reading was performed by a pediatric endocrinologist (hyperinsulinism expert) together with a nuclear medicine physician and a pediatric surgeon at the site of the PET scan. After completion of data collection, all ^18^F-DOPA and ^68^Ga-NODAGA-exendin-4 PET/CT images were reevaluated by 1 masked ^68^Ga-NODAGA-exendin-4–experienced nuclear medicine physician (expert reading). Additionally, a joint reevaluation of all images was performed by this nuclear medicine physician together with a pediatric endocrinologist highly experienced in ^18^F-DOPA PET/CT reading. All images were evaluated in terms of disease subform and, if detected, the size and location of the focal lesion. For exact localization of the focal lesion, the pancreas was divided into 6 areas based on anatomic relation to the pancreatic duct and portal vein (Supplemental Fig. 1). Images from all time points were used for interpretation.

Quantitative analysis of the ^18^F-DOPA PET/CT and ^68^Ga-NODAGA-exendin-4 PET/CT or PET/MRI scans in which focal lesions had been confirmed by histopathology was performed by a nonmasked expert. Volumes of interest (VOIs) were drawn to determine tracer uptake, expressed as SUV_max_, in different parts of the pancreas and visible focal lesions. The SUV_max_ ratios of the focal lesion or area with the highest tracer uptake (for ^18^F-DOPA PET/CT scans in which the focal lesion was not detected) and the area with the next highest tracer uptake were determined. VOIs were drawn over the head, body, and tail of the pancreas. Within these VOIs, isocontour VOIs were created consisting of the voxels with the 30% highest intensity (examples of resulting VOIs are depicted in Supplemental Fig. 2). For quantification of the dynamic PET scans, reconstructed images of the time frames from 30 to 40 min (Charité University Hospital) and from 40 to 45 min (University College London) after injection were used.

To estimate the optimal imaging time point for ^68^Ga-NODAGA-exendin-4 PET/CT, SUV_max_ ratios in reconstructed images of 5-min intervals over the imaging period from 0 to 45 min after tracer injection were determined in 4 patients with focal disease.

To evaluate image quality and correlation of imaging results with the intraoperative findings, ^18^F-DOPA PET/CT and ^68^Ga-NODAGA-exendin-4 PET/CT images of 13 patients with a detected focal lesion were rated by the involved pediatric surgeon using a questionnaire based on the Leiden surgical rating scale (supplemental materials).

### Statistical Analysis

Imaging results confirmed by histopathology were regarded as true-positives or true-negatives. Imaging results with competing histopathology results were regarded as false-positives or false-negatives. Ninety-five percent CIs for sensitivity were calculated using the Clopper–Pearson method. Interobserver variation was calculated using the Fleiss κ. SUV_max_ ratios in ^18^F-DOPA PET/CT and ^68^Ga-NODAGA-exendin-4 PET/CT scans were compared using paired-sample *t* tests. Surgeon scores of image quality were compared using Wilcoxon signed-rank tests. Statistical analyses were performed using SPSS (version 22; IBM).

## RESULTS

### Patients

We included the data of 19 CHI patients. Baseline characteristics of the patients are given in Table 1, and clinical details are in Supplemental Table 2. All patients underwent ^18^F-DOPA PET/CT and ^68^Ga-NODAGA-exendin-4 PET/CT, with a median time of 13 d (range, 4–72 d) between the procedures. On clinical reading, ^18^F-DOPA PET/CT revealed focal areas of high tracer uptake suggestive of focal lesions in 10 patients (53%), and ^68^Ga-NODAGA-exendin-4 PET/CT revealed such areas in 14 patients (74%) (Table 2). The study profile is depicted in Supplemental Figure 3.

**TABLE 1:** Patient Characteristics

Characteristic	Data
Participants	19
Age (mo)	8.3 (4.0–22.0)
Age at diagnosis (d)	7 (1.5–12)
Sex	
Female	8/19 (42%)
Male	11/19 (58%)
Genetic mutation	
Paternal ABCC8 mutation	16/19 (84%)
No or unknown mutation	3/19 (16%)
Response to medication	
Full	5/19 (26%)
Partial	14/19 (74%)

Qualitative data are number and percentage; continuous data are median and interquartile range.

**TABLE 2:** Sensitivity of ^18^F-DOPA PET/CT and ^68^Ga-NODAGA-Exendin-4 PET/CT Based on Clinical and Study Readings

Parameter	^18^F-DOPA PET/CT	^68^Ga-NODAGA-exendin-4 PET/CT
Focal lesions detected on clinical reading (*n*)	10/14 (71%)	14/14 (100%)
True-positives	10	14
False-negatives	4	0
Sensitivity* based on…		
Clinical reading	71% (95% CI, 42%–92%)	100% (95% CI, 77%–100%)
Expert reading	86% (95% CI, 57%–98%)	93% (95% CI, 66%–100%)
Joint reading	100% (95% CI, 77%–100%)	100% (95% CI, 77%–100%)

*Data are value and 95% confidence interval, calculated for cases with focal lesions only.

### Tolerability

One patient (5%) experienced vomiting after injection of ^68^Ga-NODAGA-exendin-4. In 2 patients (11%), episodes of mild hypoglycemia requiring increased glucose infusion occurred after injection of ^68^Ga-NODAGA-exendin-4. In the other patients, glucose levels were stable (>3.5 mmol/L) under regular monitoring and intravenous glucose infusion. No other adverse events occurred in any of the patients.

### Surgery

The results of both imaging procedures were used for surgical planning. Suggestive lesions were detected by clinical reading of the PET images in 14 patients. These patients underwent surgery, and the presence of focal lesions was confirmed by histopathologic evaluation. All surgically treated patients with focal lesions were cured after surgery (normalization of blood glucose levels in long-term follow-up).

Of the remaining 5 patients, in whom no focal lesion was detected by clinical reading (diffuse tracer uptake on both ^18^F-DOPA and ^68^Ga-NODAGA-exendin-4 PET/CT), 1 patient underwent near-total pancreatectomy because of an insufficient response to medication. In line with the imaging results, histopathology indicated diffuse disease in this patient. The 4 patients who did not undergo surgery (aged 10, 18, 68, and 125 mo) could be sufficiently managed with diet and medication.

Since histopathology and clinical follow-up were the reference standard, patients who did not undergo surgery were excluded from analysis.

### Diagnostic Performance

**FIGURE 1.**
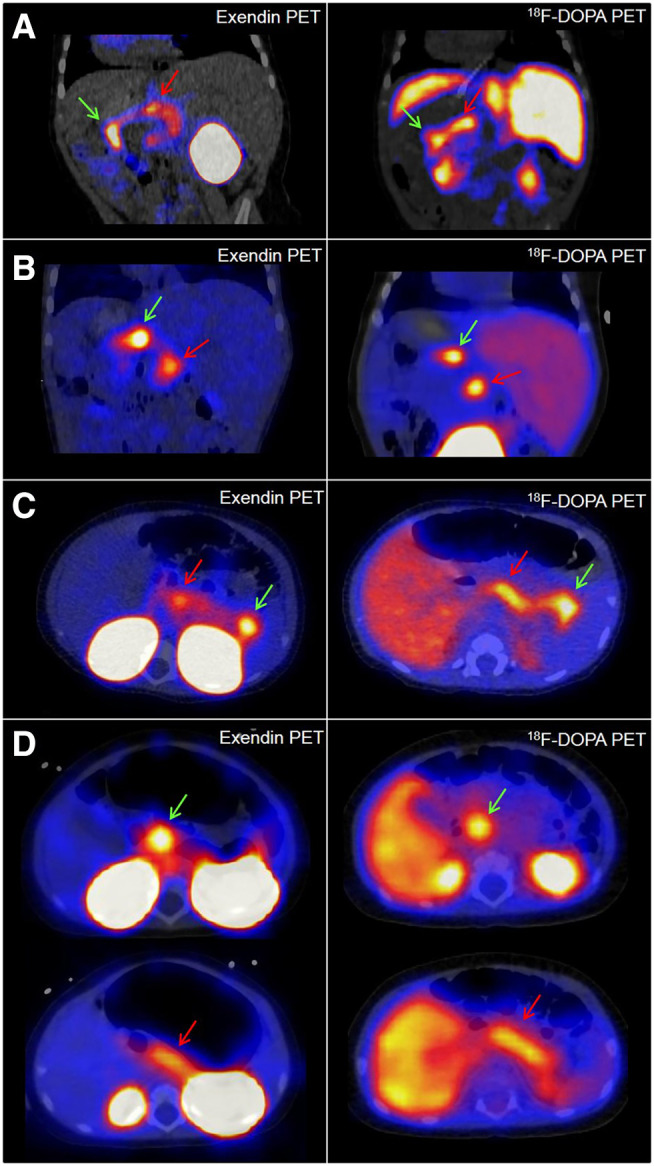
^68^Ga-NODAGA-exendin-4 PET/CT and ^18^F-DOPA PET/CT images of patients 2 (A), 4 (B), 6 (C), and 9 (D), for whom ^68^Ga-NODAGA-exendin-4 PET/CT scans were reported as focal in clinical readings whereas ^18^F-DOPA PET/CT scans were reported as diffuse. Locations of focal lesions (for ^18^F-DOPA PET/CT detected during joint readings) are indicated with green arrows. In D, focal lesion in head is indicated with green arrows, and for comparison, red arrows indicate areas with the next highest tracer uptake in all images. Presence of focal lesions was confirmed by histopathology in all 4 patients.

Imaging results are summarized in Table 2. In this patient population, ^68^Ga-NODAGA-exendin-4 PET/CT had a sensitivity of 100% (95% CI, 77%–100%) for detection of focal lesions, compared with a sensitivity of 71% (95% CI, 42%–92%) for ^18^F-DOPA PET/CT, based on clinical readings of the images. In 4 of 15 patients (27%), focal lesions were identified only using ^68^Ga-NODAGA-exendin-4 PET/CT. In these patients, the surgery planning was based solely on the results of the ^68^Ga-NODAGA-exendin-4 PET/CT. ^18^F-DOPA PET/CT and ^68^Ga-NODAGA-exendin-4 PET/CT images of these 4 patients are shown in Figure 1. On the basis of the clinical readings, ^68^Ga-NODAGA-exendin-4 PET/CT performed better for detection of focal lesions.

### Interobserver Correlation

On reevaluation of the ^18^F-DOPA PET/CT images by an expert nuclear medicine physician, 2 additional focal lesions were identified, increasing the sensitivity to 86% (range, 57%–98%). In the expert readings of the ^68^Ga-NODAGA-exendin-4 PET/CT images, 1 focal lesion was missed, decreasing the sensitivity to 93% (range, 66%–100%). Through joint reading of the images by an expert nuclear medicine physician and a pediatric endocrinologist, all focal lesions were detected on both ^68^Ga-NODAGA-exendin-4 PET/CT and ^18^F-DOPA PET/CT. Although the sensitivity of both techniques reached 100%, the interobserver agreement was higher for ^68^Ga-NODAGA-exendin-4 PET/CT than for ^18^F-DOPA PET/CT (Fleiss κ = 0.91 vs. 0.56). For ^68^Ga-NODAGA-exendin-4 PET/CT, there was almost perfect agreement, whereas for ^18^F-DOPA PET/CT, the level of agreement between the readings was only moderate. The increased sensitivity of ^18^F-DOPA PET/CT on only reevaluation of the images, together with the higher interobserver agreement in evaluation of the ^68^Ga-NODAGA-exendin-4 PET/CT images, clearly indicates a facilitated and more reliable interpretation of the ^68^Ga-NODAGA-exendin-4 PET/CT images, resulting in less equivocal results.

### Semiquantitative Analysis

**FIGURE 2.**
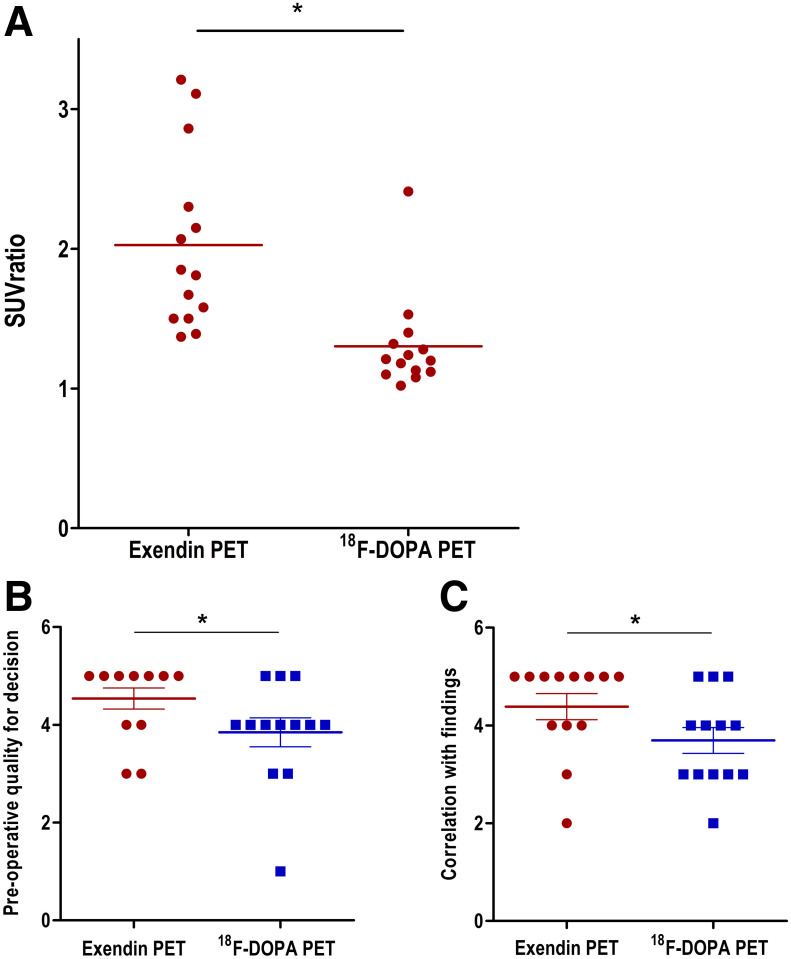
(A) SUV_max_ ratios between focal lesion and area with next highest tracer uptake. Data are given as mean ± SD, as well as individual datapoints. Scans with focal lesions identified during clinical reading are depicted in red. Scans reported to show diffuse disease are depicted in blue. (B and C) Rating scores of ^68^Ga-NODAGA-exendin-4 and ^18^F-DOPA PET/CT images by pediatric surgeons. Scores are on preoperative image quality for decision to perform surgery (B) and correlation of imaging results with intraoperative findings (C). *P < 0.05.

In patients with histopathologically proven focal CHI, the SUV_max_ ratios of the focal lesion to the area of the pancreas with the next highest tracer uptake are significantly higher in ^68^Ga-NODAGA-exendin-4 PET/CT than in ^18^F-DOPA PET/CT (2.03 ± 0.63 and 1.03 ± 0.35, respectively; *P* = 0.0026) (Fig. 2). These quantitative data show that ^68^Ga-NODAGA-exendin-4 PET/CT provides better contrast to discriminate between focal and diffuse disease and thus explains the difference in interobserver agreement levels.

Quantification of reconstructed images of 5-min intervals over the imaging period shows some variability between patients in the time point of the highest SUV_max_ ratio. SUV_max_ ratios over time are depicted in Supplemental Figure 4. For all 4 patients, the highest SUV_max_ ratio was between 30 and 45 min after tracer injection. This therefore seems to be the best time frame for ^68^Ga-NODAGA-exendin-4 PET/CT imaging.

### Surgical Ease

The influence of PET image quality on surgical ease was measured by rating of the DOPA and ^68^Ga-NODAGA-exendin-4 PET/CT images by the involved pediatric surgeons. These were all experienced surgeons who had performed more than 10 CHI surgeries during their career and more than 5 during the last 3 y. The surgeons’ rating of PET image quality showed significantly higher scores for ^68^Ga-NODAGA-exendin-4 PET/CT than for ^18^F-DOPA PET/CT regarding the decision to perform surgery (4.5 vs. 3.8, respectively; *P* = 0.025; Fig. 2B), as well as regarding correlation of the imaging results with intraoperative findings (4.4 vs. 3.7, respectively; *P* = 0.0083; Fig. 2C). Of 13 cases, the surgeons reported preferring ^68^Ga-NODAGA-exendin-4 PET/CT imaging for future CHI patients in 9 cases, versus ^18^F-DOPA PET/CT in only 1 case and no preference in 3 cases. This finding implies a better image quality for ^68^Ga-NODAGA-exendin-4 PET/CT than for ^18^F-DOPA PET/CT and a possible benefit for the surgical treatment of CHI patients.

## DISCUSSION

The results of the present study indicate that ^68^Ga-NODAGA-exendin-4 PET/CT is a promising tool for detection and localization of focal CHI, providing a high sensitivity and diagnostic accuracy. The higher interreader agreement of ^68^Ga-NODAGA-exendin-4 PET/CT than of ^18^F-DOPA PET/CT indicates superior performance of ^68^Ga-NODAGA-exendin-4 PET/CT in some patients by providing less equivocal results.

Quantitative analysis of scans of patients with histopathologically confirmed focal CHI showed significantly higher SUV_max_ ratios on ^68^Ga-NODAGA-exendin-4 PET/CT than on ^18^F-DOPA PET/CT. The higher contrast between uptake in the focal lesion and in the remainder of the pancreas on ^68^Ga-NODAGA-exendin-4 PET/CT scans enables easier detection of focal lesions. This finding explains the increased sensitivity of ^68^Ga-NODAGA-exendin-4 PET/CT based on the initial clinical reading, and the increased rate of agreement in readings of ^68^Ga-NODAGA-exendin-4 PET/CT images between clinical and expert readers, in comparison to ^18^F-DOPA PET/CT images. This increased sensitivity and agreement are of importance, especially in cases with a heterogeneous pattern of tracer uptake in the pancreas—cases that are usually difficult to diagnose (*13*).

The superior image quality of ^68^Ga-NODAGA-exendin-4 PET/CT is important for both the scan reading and the surgical procedure, since successful surgery depends on precise presurgical detection of focal CHI and the subsequent discovery and complete removal of the focal lesion intraoperatively, as reflected by pediatric surgeons’ image ratings. ^68^Ga-NODAGA-exendin-4 PET/CT could benefit the surgical treatment of CHI patients by facilitating the decision to perform surgery, as well as the intraoperative localization of the focal lesion.

In our limited population of 19 CHI patients, 4 cases of focal CHI were clinically not identified using ^18^F-DOPA PET/CT. This is a high number compared with a previous large prospective study with 50 cases and retrospective reviews of 105 and 195 cases, which reported sensitivities of 88%, 85%, and 89%, respectively, for detection of focal CHI using ^18^F-DOPA PET/CT (*13*,*17*,*18*). This result is suggestive of a high complexity in the cases in this study. An overrepresentation of such difficult cases in our study population could stem from an increased incentive to refer patients with complex and equivocal ^18^F-DOPA PET/CT imaging results for an investigative diagnostic procedure. In this population, ^68^Ga-NODAGA-exendin-4 PET/CT outperformed ^18^F-DOPA PET/CT in the clinical nonexpert setting, facilitating curative surgery without the need for further medicinal treatment or near-total pancreatectomy in 4 additional patients. ^68^Ga-NODAGA-exendin-4 PET/CT therefore had a major positive impact on the clinical management of these patients.

A limitation of the current study was the inability to exclude the possibility of missed focal lesions. All focal lesions that were identified by ^68^Ga-NODAGA-exendin-4 PET/CT were confirmed by histopathology. However, 4 patients in whom diffuse disease was indicated on both ^18^F-DOPA PET/CT and ^68^Ga-NODAGA-exendin-4 PET/CT did not undergo surgery but instead received continued medical treatment. In these patients, diffuse disease was not confirmed.

Another possible limitation of this study was the inclusion of patients from 3 centers with differences in the ^68^Ga-NODAGA-exendin-4 PET/CT acquisition procedure (Supplemental Table 1). However, the high degree of diagnostic accuracy and interreader agreement for ^68^Ga-NODAGA-exendin-4 PET/CT in all included patients shows the robustness of this technique in clinical practice.

On the basis of the data in this study, the expected optimal time frame for ^68^Ga-NODAGA-exendin-4 PET/CT imaging is between 30 and 45 min after tracer injection. In the tail of the pancreas, focal lesions that overlap the contour of the left kidney pose a diagnostic challenge with ^18^F-DOPA PET/CT, as has been described previously (*13*). Because of the high renal accumulation of ^68^Ga-NODAGA-exendin-4 PET/CT, this issue also occurs with PET/CT using this tracer. In such cases, performing additional scans at later time points could be beneficial, since uptake of ^68^Ga-NODAGA-exendin-4 PET/CT in the kidneys was shown to decrease over time in adults (*19*).

The introduction of ^18^F-DOPA PET/CT to discriminate between focal and diffuse CHI has had a major impact on the clinical approach by obviating more invasive diagnostic procedures, such as selective arterial calcium stimulation with simultaneous venous sampling or transhepatic portal venous insulin sampling, and by optimizing surgical treatment because of increased diagnostic accuracy. This study showed that ^68^Ga-NODAGA-exendin-4 PET/CT has the potential to even further improve the treatment of patients with focal CHI by improving diagnostic accuracy and certainty. This improvement could enable curative surgery in more patients and could benefit surgical planning by providing more precise and reliable preoperative images.

In addition to the better image quality of ^68^Ga-NODAGA-exendin-4 PET/CT than of ^18^F-DOPA PET/CT, another important advantage to ^68^Ga-NODAGA-exendin-4 PET/CT is the production of ^68^Ga by a generator. Because of the short half-life of ^68^Ga (68 min), it cannot be transported between centers. However, production by a generator enables on-site production of the radiotracer even at PET centers without a cyclotron, thereby enabling broad availability. Since ^18^F-DOPA is often difficult to obtain, this capability could transform the care of focal-CHI patients at such centers. In addition, the equivocal results provided by ^68^Ga-NODAGA-exendin-4 PET/CT will also enable correct image interpretation at less experienced centers. An additional important advantage to ^68^Ga-NODAGA-exendin-4 is the very low PET radiation dose to the patients, which we previously calculated to be about 4-fold lower for newborn patients than the radiation dose from ^18^F-DOPA (*19*).

## CONCLUSION

Through this study, we provided the first (to our knowledge) clinical evidence of detection and localization of focal CHI using ^68^Ga-NODAGA-exendin-4 PET/CT. These first results show that image quality is better for ^68^Ga-NODAGA-exendin-4 PET/CT than for the standard ^18^F-DOPA PET/CT, resulting in an excellent sensitivity and diagnostic accuracy, which changed the surgical management in 4 of 19 patients. Although the performance of ^68^Ga-NODAGA-exendin-4 PET/CT needs to be further assessed in a larger patient population, we believe that it has the potential to replace ^18^F-DOPA PET/CT as the primary imaging tool for detection and localization of focal CHI.

## DISCLOSURE

This work was supported by BetaCure (FP7/2014-2018, grant 602812). PET/MRI use at Charité University Hospital was supported by Deutsche Forschungsgemeinschaft (INST 335/543-1 FUGG). Martin Gotthardt is an inventor on, and holder of, the patent “Invention Affecting GLP-1 and Exendin” (Philipps-Universität Marburg, June 17, 2009). Paolo De Coppi is supported by NIHR BRC Great Ormond Street Hospital. No other potential conflict of interest relevant to this article was reported.

## Notes

KEY POINTS **QUESTION:** Is ^68^Ga-NODAGA-exendin-4 PET/CT effective for preoperative detection and localization of focal CHI? **PERTINENT FINDINGS:** ^68^Ga-NODAGA-exendin-4 PET/CT has higher clinical sensitivity and a better interobserver correlation for the detection of focal CHI than does ^18^F-DOPA PET/CT. ^68^Ga-NODAGA-exendin-4 PET/CT provided significantly higher target-to-nontarget ratios than did ^18^F-DOPA PET/CT, and pediatric surgeons rated ^68^Ga-NODAGA-exendin-4 PET/CT superior to ^18^F-DOPA PET/CT. **IMPLICATIONS FOR PATIENT CARE:** ^68^Ga-NODAGA-exendin-4 PET/CT has the potential to benefit the treatment of patients with focal CHI by improving diagnostic accuracy and certainty. This advantage could enable curative surgery in more patients and benefit surgical planning by providing more precise and reliable preoperative images.
